# The Gram-negative permeability barrier: tipping the balance of the in and the out

**DOI:** 10.1128/mbio.01205-23

**Published:** 2023-10-20

**Authors:** Claire Maher, Karl A. Hassan

**Affiliations:** 1 College of Engineering, Science and Environment, University of Newcastle, Newcastle, Australia; 2 ARC Centre of Excellence in Synthetic Biology, Macquarie University, Sydney, Australia; Case Western Reserve University School of Medicine, Cleveland, Ohio, USA; University of Guelph, Guelph, Ontario, Canada

**Keywords:** Gram-negative bacteria, cell envelope, antibiotic resistance

## Abstract

Gram-negative bacteria are intrinsically resistant to many antibiotics, due in large part to the permeability barrier formed by their cell envelope. The complex and synergistic interplay of the two Gram-negative membranes and active efflux prevents the accumulation of a diverse range of compounds that are effective against Gram-positive bacteria. A lack of detailed information on how components of the cell envelope contribute to this has been identified as a key barrier to the rational development of new antibiotics with efficacy against Gram-negative species. This review describes the current understanding of the role of the different components of the Gram-negative cell envelope in preventing compound accumulation and the state of efforts to describe properties that allow compounds to overcome this barrier and apply them to the development of new broad-spectrum antibiotics.

## INTRODUCTION

Antibiotic resistance is a rapidly growing global health crisis. Between 2017 and 2020, a 15% rise in antibiotic resistance was observed for bloodstream infections caused by *Escherichia coli*, *Salmonella* spp., and *Neisseria gonorrhoeae* (1). These increasing resistance rates are driving the usage of “last resort” antibiotics, and in some cases, bacteria have been identified with resistance to even these compounds, leading to essentially untreatable infections (2). In 2019, an estimated 1.27 million deaths globally were directly attributable to antibiotic resistance (3). One estimate predicts that if the current resistance trajectory continues, antibiotic resistance will be the leading cause of death by 2050 (4). A multifaceted strategy is required to combat this complex threat, and a key component of this is the development of new antibiotics capable of treating resistant bacterial infections.

Whereas some antibiotics have broad-spectrum activity, others are effective only against particular groups of bacteria due to intrinsic resistance in other groups. Gram-negative bacteria are intrinsically resistant to many antibiotics due to the low permeability of their cell envelopes. Of the 1.27 million deaths attributable to antibiotic resistance in 2019, the majority were caused by six pathogens, four of which were Gram-negative species (*E. coli*, *Klebsiella pneumoniae*, *Acinetobacter baumannii*, and *Pseudomonas aeruginosa*) (3). Gram-negative species also make up the highest priority (critical) pathogens for new antibiotic development as determined by the World Health Organization (5). A lack of detailed understanding of how different components of the cell envelope act to exclude or to facilitate the passage of antibiotics has been identified as a key barrier to the rational design of new antibiotics with Gram-negative efficacy (6).

## THE GRAM-NEGATIVE CELL ENVELOPE

**Fig 1:**
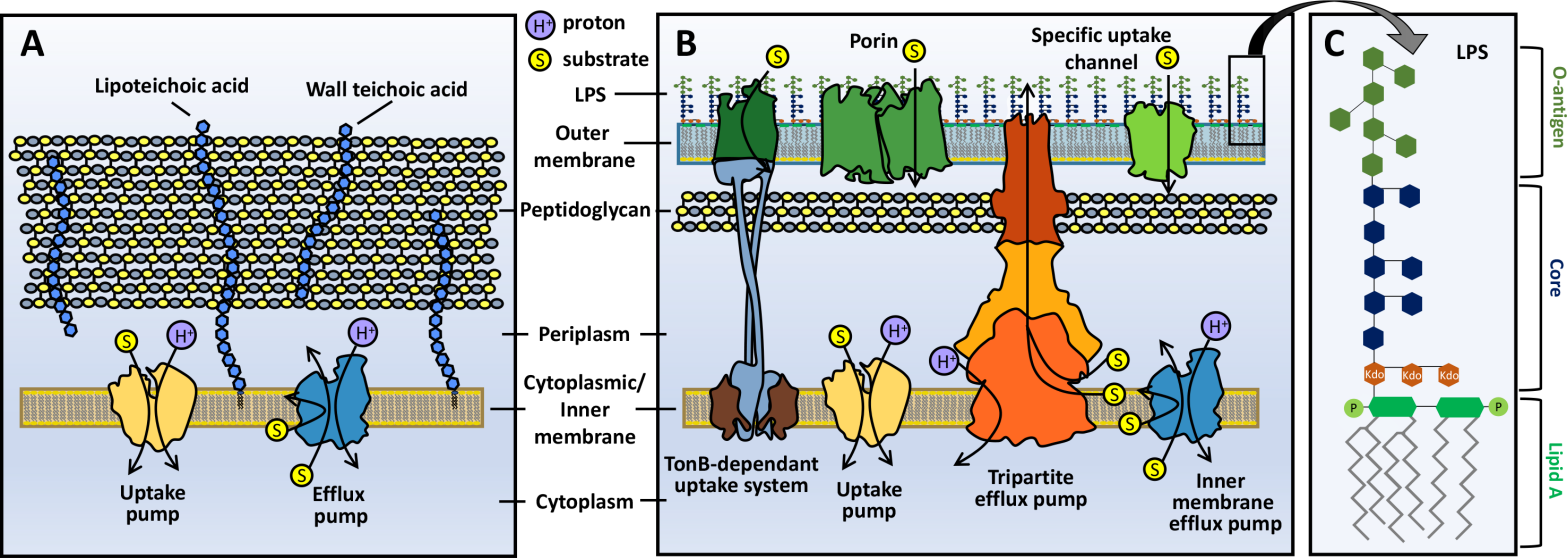
Simplified depictions of the cell envelopes of Gram-positive (**A**) and Gram-negative (**B**) bacteria. Both envelope types contain a cytoplasmic membrane (often termed the inner membrane in Gram-negatives) consisting of phospholipids, surrounded by a peptidoglycan layer. In Gram-positives, the peptidoglycan layer is thicker and contains teichoic acids bound to the peptidoglycan only (wall teichoic acids) or peptidoglycan and the cell membrane (lipoteichoic acids). Gram-negative species possess a second, outer membrane that has an inner leaflet composed mainly of phospholipids and an outer leaflet composed mainly of lipopolysaccharides (LPS). The LPS molecules (**C**) consist of three components: lipid A, a variable oligosaccharide core, and a variable oligosaccharide chain termed O-antigen. As well as lipids, both membranes also contain proteins. The cytoplasmic membrane contains uptake proteins and efflux pumps, which in Gram-negatives can be either single component pumps spanning the inner membrane or tripartite pumps spanning both membranes. The outer membrane of Gram-negatives also contains specific uptake channels and general porins.

The structure of the cell envelope is an important factor in defining bacterial groups. The Gram-positive cell envelope consists of a cytoplasmic membrane, which inhibits the passage of charged and hydrophilic molecules, surrounded by a thick peptidoglycan cell wall, which endows the cell with rigidity but imposes little restriction on the passage of small molecules (<50 kDa limit for globular hydrophilic compounds that do not bind to the cell wall) (7). Gram-positive cell envelopes also contain large amounts of anionic polymers termed teichoic acids, which may be coupled to either the peptidoglycan (wall teichoic acids) or the cell membrane (lipid teichoic acids) and give the cell surface an overall negative charge (Fig. 1A) (8).

Gram-negative bacteria also possess a cytoplasmic membrane, which is surrounded by a thinner layer of peptidoglycan, and a secondary, outer membrane (OM) that provides stability to the cell and acts as a permeability barrier (Fig. 1B). Although lipid compositions vary among individual species, the Gram-negative inner membrane (IM) typically has a higher concentration of the zwitterionic phospholipid phosphatidylethanolamine, and often of zwitterionic phospholipids in general, than the Gram-positive cytoplasmic membrane, which may instead have higher proportions of anionic lipids such as phosphatidylglycerol and cardiolipin (9–12). Similar to Gram-positive bacteria, the IM inhibits the passage of charged and hydrophilic compounds. The Gram-negative OM is an asymmetrical lipid bilayer, with a phospholipid inner leaflet similar to the IM and an outer leaflet composed primarily of lipopolysaccharides (LPS), which make the OM less permeable to hydrophobic compounds than a typical lipid bilayer (Fig. 1C).

Differences in physiochemical properties have been identified between antibiotics with broad-spectrum activity compared to Gram-positive-specific compounds, as well as between Gram-negative antibiotics with different modes of passage through the IM (13). Some antibiotics—typically more hydrophobic compounds such as chloramphenicol and fluoroquinolones—can cross the IM through passive diffusion. Similarly, many Gram-positive-specific antibiotics with cytoplasmic targets, such as macrolides, would be likely to cross the Gram-negative IM through diffusion if they were able to pass across the OM barrier. The passage of some compounds across the IM may be aided by electrochemical potentials, such as the proton motive force (14). For example, there is a general consensus that aminoglycoside uptake occurs in two energy-dependent steps. First, slow movement across the IM occurs, which is reliant upon membrane potential. Once inside the cytosol, aminoglycosides bind to ribosomes and induce protein mistranslation. These misfolded proteins incorporate into and disrupt the membrane, allowing further aminoglycoside penetration and eventually leading to cell death (15, 16).

Antibiotics such as cycloserine, negamycin, and fosfomycin utilize active, carrier-dependent pathways to cross the IM, although this is rare, likely due to the non-essential nature of such transporters making the rapid development of resistance possible (17, 18). In fact, cycloserine and negamycin are not limited to uptake through carriers and, in certain environmental conditions, are able to cross the IM through passive diffusion. In contrast, while fosfomycin resistance through loss of uptake transporters arises with high frequency *in vitro* in *E. coli*, these rates are not reflected clinically. Strains with transporter mutations show poor growth in the presence of fosfomycin, which is predicted to be sufficient to prevent the establishment of resistant infections *in vivo* (19, 20). This indicates that in some cases, carrier-dependent entry is a viable pathway through the IM for clinically effective antibiotics.

Both membranes contain proteins that allow the passage of nutrients and other required molecules into the cell. In the OM, these include specific uptake channels and general porins, which can function as entry routes for hydrophilic and amphiphilic antibiotics. Efflux pumps are localized to the IM or span the IM and OM. These pumps export compounds from the cytoplasm to the periplasm, or from the periplasm out of the cell, respectively, and work synergistically with the two membranes to prevent the accumulation of many compounds in Gram-negative cells that are effective against Gram-positives (21–23).

Despite the complexity of these interactions and the variations in permeability between different Gram-negative species, progress has been made in recent years toward developing an understanding of the characteristics of compounds that improve their accumulation in Gram-negatives and applying these to the development of new compounds. This review aims to give an overview of these recent advances.

## OUTER MEMBRANE LIPOPOLYSACCHARIDE AND LIPOOLIGOSACCHARIDE

The LPS outer leaflet of the Gram-negative OM is the major barrier to small molecule permeability, but there is growing evidence that variations in its precise composition have a significant influence on permeability. LPS typically consists of three components—lipid A, consisting of four to seven acyl chains attached to a variable phosphorylated disaccharide; a variable phosphorylated oligosaccharide core; and a repeating oligosaccharide chain of variable length termed O-antigen (Fig. 1). For some bacterial species, including *A. baumannii* (24) and *Neisseria meningitidis* (25), an O-antigen ligase has not been identified, and it is believed that these species lack O-antigen, producing molecules consisting of only lipid A and the core, termed lipooligosaccharides (LOS), and non-covalently attached polysaccharides as a capsule. Divalent cations (Mg^2+^ and Ca^2+^) act as a bridge between the negative charges of lipid A and the core of adjacent LPS and also form crucial interactions with OM proteins. This bridging, along with the predominately saturated nature of the lipids, tightly packs the LPS molecules, making the OM more rigid than a typical phospholipid bilayer and slowing the diffusion of hydrophobic compounds by up to two orders of magnitude in comparison (26).

The asymmetry of the OM is critical to its barrier function and Gram-negative species possess multiple systems with overlapping roles to maintain the LPS/LOS outer leaflet. While phospholipids from the inner leaflet may flip into the outer leaflet at low rates during normal growth, OM damage can cause the loss of LPS and the incorporation of phospholipid into the outer leaflet in its place (27–29). Phospholipids and LPS do not readily mix and instead cluster together to form regions in the membrane that are more permeable to harmful compounds (30, 31). Gram-negatives possess various mechanisms to remove mislocalized phospholipids and maintain asymmetry. The OM protein PagP transfers a palmitoyl acyl chain from a mislocalized phospholipid to phosphatidylglycerol or lipid A of LPS, resulting in a lysophospholipid by-product (32–35). These lysophospholipids can then be transported to the IM for recycling, or they may be degraded by PldA, which also degrades outer leaflet phospholipids (36, 37). Further, the acylation of LPS and phosphatidylglycerol by PagP also decreases OM permeability (32, 35, 38). Alongside these degradation pathways, there is evidence to suggest that the mla system transports phospholipids from the outer leaflet of the OM to the IM (39), although the direction of phospholipid transport remains a point of contention [as recently reviewed by Abellon-Ruiz (40)].

LPS molecules are diverse among bacterial strains, and bacteria possess a range of mechanisms to modify them in ways that alter membrane permeability, typically in response to environmental conditions or stresses, including antibiotics. Modifications to lipid A generally involve modifying the net charge of the molecule through the addition or removal of sugars, phosphates, and other groups, as well as alterations to the number and length of acyl chains (41–45). Similar modifications that reduce the net negative charge of the core are also found (46, 47). Although providing resistance to some compounds, a reduction in LPS negative charge may be highly detrimental to bacterial fitness (48, 49), emphasizing the careful balance bacteria must strike when altering the OM to decrease antibiotic permeability. As well as affecting OM surface charge, such modifications can also alter properties such as the fluidity and packing of LPS and their interactions with OM proteins. These changes can result in increased resistance to cationic antimicrobial peptides (CAMPs) and some large, hydrophobic compounds (32, 50, 51). In some circumstances, the complete loss of LPS/LOS in the outer leaflet of the OM has been identified as an adaptive resistance mechanism. In Gram-negative species including *A. baumannii* and *E. coli*, prolonged exposure to colistin, a polymyxin that targets LPS/LOS, can lead to the loss of LPS/LOS through mutations in lipid A biosynthesis. This can provide colistin resistance but at the cost of increased susceptibility to a range of other compounds (52–54).

O-antigen is the most variable component of LPS and, indeed, one of the most variable components of bacterial cells. This variability has made O-antigen useful for classifying and identifying Gram-negative strains by O-antigen serotyping. It is theorized that this diversity has arisen from selective pressures such as host immune systems and predation by bacteriophages and protists (55, 56). Mutants lacking O-antigen often display increased sensitivity to detergents and hydrophobic antibiotics, suggesting that O-antigen reduces the permeability of these compounds (57–59). These mutants can show a reduction in OM proteins and an increase in glycerophospholipids, which may also account for changes in permeability (59, 60). The presence and length of O-antigen are also likely to affect membrane fluidity and mechanical strength, which further influences the permeability of some compounds (61–63). Individual LPS modifications could work cooperatively to reduce permeability. For example, in response to certain environmental stressors, *Salmonella enterica* serovar Typhimurium modifies lipid A through the addition of a hydroxyl group, a palmitoyl chain, and an aminoarabinose sugar. Molecular dynamics simulations found that the addition of palmitoyl resulted in a thickened OM and not only increased LPS packing but also weakened hydrogen bonding between LPS molecules (51). Hydroxylation contributed a minor increase in hydrogen bonding but matched the degree of bonding lost by palmitoylation. Therefore, hydroxylation may, in this case, serve to offset the negative impacts of palmitoylation.

## PORINS AND OTHER OM UPTAKE PROTEINS

Proteins constitute approximately 50% of the mass of the OM, and a large proportion of these are porins (64, 65). Porins are β-barrel proteins that allow small hydrophilic and amphiphilic molecules, including antibiotics, to cross the OM. Differences in porin content are typically considered the largest contributor to the distinct permeability profiles of different Gram-negative bacteria.

In non-specific porins such as OmpF and OmpC from *E. coli*, the size of the channel is primarily dictated by loop L3 of the protein, which folds into the barrel and reduces the area for substrate passage (66, 67). Loop L3 contains several acidic amino acid residues opposite a series of basic residues in the pore channel, generating a transverse electric field (68). A similar structure has been observed for porins from several other species (69, 70). Electrostatic interactions in the porin channel may play a larger role in permeability than pore size, with more flexible molecules better able to alter their conformation to align their dipole with the electrostatics of the channel (71, 72). In contrast to general porins, specific porins typically have multiple loops folded into the pore channel, creating a narrower passage for substrates (73, 74). OmpF is generally considered to have a size exclusion limit of ~600 Da, although this was recently challenged by a study that found that a 692-Da antibiotic was able to pass through OmpF by adopting a more compact conformation (75). Species such as *P. aeruginosa* and *A. baumannii* have large numbers of specific porins, which have size exclusion limits of ~200 Da, and their estimated OM permeabilities are 1%–8% of that of *E. coli*, which is ascribed in large part to their different porin contents, as well as differences in efflux pump content (discussed below) (76–78).

Alterations to porin content have been associated with resistance adaptations in clinical isolates from a range of species. Mutations in individual porins can lead to structural changes that narrow the pore diameter and/or alter its electrical field, reducing the permeation rates of antibiotics, such as carbapenems in *E. coli* and ampicillin in *N. meningitidis* (79, 80). Downregulation of porin expression and porin exchange have also been observed as resistance strategies (81–84). It remains unclear in cases where major porins are lost or expressed at significantly lowered levels whether the vacant membrane space is filled by other β-barrel proteins or LPS/phospholipids, but recent work has suggested that LPS levels remain consistent in *E. coli* in the absence of the porins OmpF, OmpC, and LamB (30). Clinically, alterations to porin content commonly co-occur with other resistance mechanisms, including efflux pump and/or β-lactamase induction, emphasizing the synergistic nature of the cell envelope and other resistance mechanisms (85–88). An expanding body of research is investigating the organization of proteins, phospholipids, and LPS in Gram-negative outer membranes (30, 89). This work will provide insight into processes of outer membrane assembly and could present novel avenues for targeting or bypassing the outer membrane (89).

Other OM proteins may also act as antibiotic uptake pathways. The 1,450 Da antibiotic vancomycin is able to move through mutants of TolC, the OM component of the AcrAB-TolC efflux pump in the presence of active AcrAB in *E. coli* (90). A number of natural antibiotics, such as albomycin and salmycin, are conjugated with siderophores, allowing them to enter cells through TonB-dependent siderophore uptake proteins in the OM (Fig. 1), such as FhuA in *E. coli* (91). Synthetic siderophore conjugates have also been designed utilizing the same principle, with cefiderocol being one of the first of these to be approved for clinical use in 2019 and many others at various stages of clinical development (92–95). Sugar-antibiotic conjugates that utilize specific uptake systems, such as maltodextrin conjugates that utilize the *E. coli* transporter LamB, are also being explored (96).

## EFFLUX PUMPS

As well as navigating two membranes, to be effective against Gram-negative bacteria, antibiotics must also effectively evade efflux. All bacteria possess efflux pumps, which actively pump toxic compounds, including antibiotics, out of the cell, preventing accumulation at intracellular target sites and leading to resistance (Fig. 1). Many of these pumps are highly promiscuous, transporting a wide range of structurally diverse compounds. As such, increased expression of a pump selected by a single antibiotic can increase the host cell’s tolerance to a range of other unrelated compounds. Overexpression of efflux systems contributes to multidrug resistance in major Gram-negative pathogens such as *P. aeruginosa*, *A. baumannii*, and *E. coli* (97–99).

Gram-negative efflux pumps may be single-component or tripartite transporters (Fig. 1B). Single-component transporters are located in the IM and transport compounds from the cytoplasm to the periplasm or from the inner to the outer leaflet of the IM. Most tripartite pumps capture compounds from the periplasm and/or outer leaflet of the IM and expel them into the extracellular space (Fig. 1B). Tripartite pumps consist of an IM protein, an OM channel, and a periplasmic adaptor protein. Seven distinct families of transport proteins are known to include efflux pumps (100). Tripartite pumps in the resistance-nodulation-cell division (RND) superfamily are considered the most significant in terms of drug resistance capabilities in Gram-negative bacteria, due to their high substrate promiscuity and ability to export compounds into the external medium. Strains of *P. aeruginosa* may encode more than 10 RND multidrug efflux pumps (101), compared to six in most *E. coli* strains (102). This could be partly responsible for the low accumulation of many compounds in *P. aeruginosa* compared to *E. coli* (alongside differences in porin content) (103, 104). IM exporters are generally less clinically significant, since it is not likely that high-level expression of any one single component IM pump will shift resistance across breakpoints. However, it has been shown that the inactivation of multiple IM pumps can render the major RND pump AcrAB-TolC in *E. coli* essentially ineffective, highlighting cooperation between IM and tripartite pumps (21).

Despite their substrate promiscuity, there are some general chemical features that may make compounds more or less likely to be subject to efflux. In *E. coli*, studies of RND pumps have suggested that molecules with high hydrophobicity (clogD 1–5) and planar, unbranched, and elongated molecules are more likely to be effectively effluxed (105). This may be due to these molecules forming looser interactions with the deep binding pocket of AcrB, which have been shown in molecular dynamics simulations to facilitate extrusion (106). Similar studies of MexB from *P. aeruginosa* suggested that compounds that are less likely to be efflux substrates have a reduced preference for the deep binding pocket (107). Another study found that highly hydrophilic (clogD < 0) and low molecular weight compounds, and high molecular weight and zwitterionic compounds, are less likely to be effluxed by RND pumps (108).

Since molecules must pass across the OM to be recognized by efflux pumps, OM selectivity must also be considered when assessing efflux pump function in whole cells. In this light, Zgurskaya and colleagues recently provided new insight into the substrate preferences of RND pumps using OM-deficient and efflux-deficient strains of several pathogens. With this approach, they were better able to assess the potential for efflux pumps to recognize a broad range of compounds, including poorly OM-permeable antimicrobials, and found that even large antibiotics could be affected by RND efflux, including compounds previously thought not to be substrates (109). Work on the OM-deficient and efflux-deficient strains is discussed further below.

Another factor that may influence the likelihood of efflux via an RND transport protein is subcellular localization. RND pumps capture substrates from the periplasm and/or outer leaflet of the IM. Most small molecules important to the cell are unlikely to dwell in these locations for extended periods of time; e.g., useful metabolites may be promptly transported into the cytoplasm by IM uptake permeases upon entry into the periplasm. Therefore, the opportunity for RND pumps to encounter such compounds is reduced. Additional complexity into the roles of efflux pumps in preventing antimicrobial accumulation comes from the timing of their expression. Recent studies have shown that pump expression can be responsive to growth rates and growth phase (110–112). This work demonstrates the importance of environmental context in assessing the real significance of efflux in resistance.

## ARE THERE PHYSICOCHEMICAL PRINCIPLES THAT COULD GUIDE GRAM-NEGATIVE ANTIBIOTIC DEVELOPMENT?

The lack of data about how components of the Gram-negative cell envelope prevent compound accumulation has been identified as a key barrier to the rational development of new Gram-negative antibiotics (113). However, recent efforts have been made to develop generalized guidelines for Gram-negative antibiotic design, and in some cases, such guidelines have been successfully applied to convert Gram-positive antibiotics into broad-spectrum agents (114, 115).

In 2016, Takrouri and colleagues developed guidelines based on published data for factors that improved compound accumulation in Gram-negatives (114). These described low molecular weight (ideally <400 Da), high polarity (clogD < 1), and a charged character with a bias toward being zwitterionic at physiological pH as being important for activity against Gram-negatives, although not sufficient. These guidelines were applied to convert analogs of a class of Gram-positive-specific antibiotics into a compound with broad-spectrum activity, although this was done at the sacrifice of potency (114).

Following this, another set of guidelines was developed utilizing data from whole-cell accumulation studies in *E. coli* (116). These were termed the eNTRy rules, referencing the characteristics that were associated with improved accumulation: the presence of a non-sterically encumbered ionizable nitrogen (particularly a primary amine), low three-dimensionality (globularity ≤ 0.25), and relatively high rigidity (≤5 rotatable bonds). These guidelines were used to successfully convert the Gram-positive-specific antibiotic deoxynybomycin into one with Gram-negative activity through the addition of a primary amine (115). This improved accumulation in *E. coli*, *A. baumannii*, *K. pneumoniae*, and *Enterobacter cloacae*, although it did not improve efficacy against *P. aeruginosa*. These results indicated that these rules have some broader applicability outside of *E. coli*, but they are not universal to all Gram-negatives. The eNTRy rules were subsequently utilized to develop a number of other compounds for use against Gram-negatives, including a fatty acid synthesis inhibitor (117) and a riboswitch inhibitor (118). There is also evidence that some antibiotic adjuvants such as OM permeabilizers also adhere to the eNTRy rules and that such permeability rules may also be useful in the development of such compounds (119).

As shown in the work described above, cytoplasmically targeted Gram-positive antibiotics may be excellent leads for Gram-negative antibiotic development because they possess the ability to cross a bacterial cytoplasmic membrane. In contrast, compounds tested using *in vitro* biochemical assays may not. Indeed, a lack of whole-cell antibiotic activity from compounds identified through biochemical screens has historically been an issue in the development of antibiotics, leading to a move toward whole-cell screening approaches (120). As such, OM permeability guidelines might be most successfully utilized in the conversion of compounds active against Gram-positives or efflux- or OM-deficient Gram-negative strains, rather than compounds tested in *in vitro* biochemical assays alone.

One of the key features identified by the eNTRy rules to promote compound accumulation was a primary amine. The addition of a primary amine has been determined to improve compound accumulation in *E. coli* by improving uptake through porins by enabling better alignment of the dipole moment of the developed compounds to the electrical field of OmpF (121, 122). This modification likely functions in a similar way in other species where primary amines are effective in improving accumulation (121, 122). Similarly, the alignment of the restrictions for molecular weight/globularity with the requirements for porin passage suggests that the eNTRy rules may be primarily describing the qualities of a compound that promote uptake through porins. Indeed, a scoring function recently developed to predict permeability through porins specifically utilizes charge and dipole as well as minimal projection area to assess compounds, similar to the characteristics described in Gram-negative accumulation guidelines (123). In the study, the correlation between predicted influx through porins and their whole-cell accumulation was found to be high, emphasizing the key role of porins in Gram-negative permeability.

The contribution of porins to compound accumulation may be more significant in some species than in others. A recent study in *P. aeruginosa* that deleted all confirmed and putative porins in the species found only minor differences in susceptibility to a range of different antibiotics (124). The one exception was a moderately increased resistance to the carbapenems meropenem and imipenem, both of which are known to specifically utilize the porin OprD for entry into *P. aeruginosa* (125). These results suggested that porins are a minor pathway for antibiotic uptake in *P. aeruginosa*, in contrast to what has been observed for species like *E. coli*, and effective antibiotics should be able to pass through the OM lipid bilayer. This example speaks to the need for species-specific rules for compound permeation.

Recently, a machine learning approach identified only weak correlations between physiochemical properties such as molecular weight and rigidity and accumulation (126). This is an example of a broader issue of conflicting findings and difficulties establishing clear relationships between physiochemical features and permeability that have been encountered in attempts to study compound accumulation. The study found that specific chemical moieties are better predictors for accumulation than basic physiochemical parameters like those that have traditionally formed accumulation guidelines (126), a finding that has been echoed in other studies (127, 128). The findings not only supported the significance of primary amines but also found that other amines, as well as thiophenes and halides, improved compound accumulation through unknown mechanisms (126). The identification of specific chemical groups beyond amines that promote accumulation is beneficial as it allows for a broader scope of possible chemical modifications in drug design, where the addition of a primary amine may not always be practicable due to limitations of compound structure or activity. The inability of physiochemical properties to adequately describe compound accumulation in all circumstances is likely due to the complexity, variation and adaptability of Gram-negative cell envelopes.

While, in some cases, the role of certain characteristics and chemical moieties in improving accumulation has been elucidated, the complex interaction of forces at the cell envelope often obscures the specific factors preventing high compound accumulation, which makes rational chemical design to overcome these barriers more difficult. For many chemical characteristics, their role in accumulation is not at all understood. Thus, a method for identifying the contributions of these different forces is required. One technique that has been applied to aid in this has been transposon-directed insertion site sequencing (TraDIS). This technique was used to examine and compare the accumulation of the β-lactams ampicillin and benzylpenicillin in *E. coli*, which have identical chemical structures except for the presence of an amine group in the former (129). The results of this study not only supported the well-established role of primary amines in improving compound uptake through OmpF but also suggested that the primary amine in ampicillin may be influencing compound efflux through AcrAB. This work demonstrates the potential of TraDIS to identify specific genes involved in antibiotic accumulation and determine potential uptake mechanisms for compounds.

Another recently developed tool to aid in understanding the complexities of drug accumulation is a kinetic model of Gram-negative membranes incorporating both influx and efflux (130). This model predicted a non-linear relationship between external compound concentration and internal accumulation based on two parameters: an efflux constant accounting for the efficiency of active efflux and a barrier constant accounting for the rate of influx. The model identified a bifurcation in the kinetics of drug accumulation that was controlled by the barrier constant. When the constant was less than 1, i.e., diffusion rates through the OM were high, efflux became saturated and compounds accumulated in the cell. When the constant was above 1, the rate of influx could not overcome the rate of efflux, and the concentration of the compound in the cell was low, potentially below the efficacy level of the compound and thus preventing its activity (130). The latter situation is particularly important for hydrophobic compounds, which saturate the OM at lower concentrations than more hydrophilic compounds that are able to pass through porins.

The primary kinetic accumulation model was subsequently adjusted to account for more factors influencing accumulation. When the model was adjusted to account for multiple transporters across both membranes, it indicated that transporters are additive when present in the same membrane and synergistic when present in different membranes (131), similar to the conclusions of previous experimental studies (22). Active efflux and limited diffusion were synergistic only when they occurred across the same membrane (130). Efflux rates were also found to be linked to potency, with cells being better protected against more toxic compounds. The predictions of the most recent version of the model match *in vivo* data relatively closely, with predicted MIC values within threefold of experimental data (131). One assumption was made that improved the performance of the model, which was necessary due to the lack of correlation between IC50 and intracellular accumulation data. This suggested that prolonged compound exposure was doing greater damage to the cell than would be expected based on accumulation levels. The underlying mechanism for this has not yet been identified.

In cases where compounds do not accumulate, the rate of accumulation could be improved either by increasing permeability across the OM or by decreasing the rate of efflux. Which of these is the better solution varies between classes of compound and determining which path to pursue in modifying drugs to improve their Gram-negative accumulation is difficult. One method has been developed to separate the contributions of the different forces, which are efflux- and OM-deficient strains. Krishnamoorthy and colleagues developed a hyperporination system that utilizes a siderophore uptake transporter that has been modified to form a large ~2.4 nm pore in the OM that allows for the non-selective passage of compounds (132). When this protein is expressed at high levels, the OM barrier is effectively removed. In combination with efflux-deficient strains with different combinations of efflux pump deletions, the impacts of the OM and efflux on the accumulation of different compounds can be elucidated more clearly. As mentioned above, Zgurskaya and colleagues used this system to show that even large antibiotics could be affected by RND efflux, but these compounds are generally excluded from cells by the OM (109). Using these systems, a diverse set of compounds have been organized into other groups based on the contribution of the OM and efflux to their accumulation levels. Studies in different species with these backgrounds have found that different species follow the same general principles defined by the accumulation model with regard to bifurcation of kinetics and synergy between membranes, but that reactions to different compounds were species-specific (109). In *P. aeruginosa*, characteristics including the shape, flexibility, and dipole moment were identified as useful descriptors for compounds that could cross the OM barrier. In *E. coli*, electrostatic interactions and rigidity were more useful. These differences likely reflect differences in porin composition and usage between the two species. The effects of efflux deficiency and hyperporination were observed to be mechanistically independent from each other, with the two barriers filtering compounds based on different properties, and, in the case of *E. coli*, these properties appeared to be orthogonal. Lipophilicity, a number of non-aromatic rotatable bonds and partial positive charges were identified as useful parameters for assessing efflux efficiency (133).

In recent work, Cox and colleagues developed a highly efflux-deficient strain of *E. coli* by inactivating all (35) of its known or putative efflux pumps (134). The team additionally incorporated the hyperporination system described above into this strain to generate a useful platform to investigate efflux pump resistance function. By expressing targeted efflux pumps, alone or in combination, this system has helped to define the major efflux systems in *E. coli* with respect to substrate range (134) and shown cooperative activities between single component IM pumps and tripartite efflux pumps that have overlapping substrate specificities (135).

## CONCLUSIONS

With the rapid rise of antibiotic resistance around the world, there is an urgent need for the development of new antibiotics and other treatments that can combat the growing crisis. In Gram-negative bacteria in particular, development pipelines for these new compounds will be greatly aided by a detailed knowledge of how compounds are able to penetrate into and accumulate in cells. An improved understanding of the specific uptake pathways of diverse compounds will be beneficial not only in the design of new compounds but also in understanding the synergistic and antagonistic relationships between different antibiotic and antibiotic/adjuvant compounds, enabling more effective combination therapies. Beyond antibiotic resistance, this foundational accumulation of knowledge will also be beneficial in areas such as industrial biotechnology and bioremediation to aid in the uptake and/or efflux of industrially important compounds across bacterial cell envelopes.

In the past decade, guidelines for compound accumulation have been developed and applied to successfully design broad-spectrum antibiotics (114, 115). Currently, the accumulation guidelines that are available primarily describe chemical features that promote uptake through porins. The exploration of alternative pathways of uptake, such as siderophore receptors and sugar transporters, is a promising avenue of research that has yet to be fully explored (136). While the uptake systems for these “Trojan horse” antimicrobials will likely need to be essential to prevent the rapid development of resistance, the success of compounds such as cefiderocol and fosfomycin is encouraging (137, 138).

Current guidelines of the physiochemical properties that promote small molecule accumulation in Gram-negative bacteria have primarily been developed in *E. coli*, although, in some cases, they have been demonstrated to have some broader applicability across other Gram-negative species (115, 117, 118). However, this work has also highlighted the need for species-specific and even strain-specific understandings of permeability due to the impact of variations in porin, efflux pump, and LPS content. The evidence to date indicates that there is unlikely to be a single set of accumulation guidelines that are applicable to all species, and more research remains to be done in non-model organisms to develop these species-specific accumulation guidelines. Furthermore, recent work has demonstrated differences in levels of accumulation depending on growth rate and growth phase (110–112), demonstrating the importance of understanding the balance of uptake/efflux in different environments when designing clinically effective antimicrobials.

While understanding is still lacking in a number of areas, the successes that have already been achieved through increasingly detailed knowledge of the accumulation of diverse compounds demonstrate the future potential for the development of effective Gram-negative drug development pipelines to combat the antibiotic resistance crisis.
